# Association Analysis of rs1695 and rs1138272 Variations in *GSTP1* Gene and Breast Cancer Susceptibility

**DOI:** 10.31557/APJCP.2020.21.4.1167

**Published:** 2020-04

**Authors:** Amir Farmohammadi, Vahid Arab-Yarmohammadi, Ramin Ramzanpour

**Affiliations:** 1 *Student Research Committee, Isfahan University of Medical Sciences, Isfahan,*; 2 *Cellular and Molecular Biology Research Center, Health Research Institute, Babol University of Medical Sciences, Babol, Iran. *

**Keywords:** Breast cancer, genetic variation, rs1695, rs1138272, structural analysis

## Abstract

**Background:**

The glutathione S transferases P1 (*GSTP1*) is one of the common type of the GSTs family. This gene has several genetic polymorphisms that the rs1695 and rs1138272 are the most common variations in this gene. This study aimed to examine the association of these genetic variations with breast cancer risk which was followed by bioinformatics analysis.

**Materials and Methods:**

In a case-control study, 200 participants including 100 women with breast cancer and 100 healthy women were enrolled. After blood sample collection and DNA extraction, the total genomic DNA was extracted from this sample. The SNPeffects online software was employed to evaluate the effects of rs1695 genetic variation on the GSTP1 protein structure.

**Results:**

Our data revealed that there is a significant association between rs1695 genetic variation and the risk of breast cancer in homozygote (OR= 3.1532, 95%CI= 1.1072 to 8.9798, p= 0.0315) and allelic (OR= 1.6098, 95%CI= 1.0577 to 2.4500, p= 0.0263) genetic comparisons. This despite the fact that the rs1138272 polymorphism was not associated with breast cancer risk. Our bioinformatics analysis based on WALTZ output showed that the rs1695 polymorphism reduces the amyloid propensity of the GSTP1 enzyme (dWALTZ= -228.00).

**Conclusions:**

Based on our findings, the rs1695 genetic variation is a genetic risk factor for breast cancer and it could be considered as a biomarker for screening of susceptible women.

## Introduction

According to the studies, breast cancer has been recently one of the commonest types of cancer with continuous prevalence throughout the world. Moreover, this cancer is the main cause of cancer-associated deaths among women (Pedraza et al., 2012). Considering the previous investigations, an annual diagnosis of approximately 1.15 million patients with breast cancer diagnosed has been reported, with the maximum occurrence of breast cancer in the USA and Europe (Siegel et al., 2012; Song et al., 2016). However, breast cancer prevalence is quickly increasing in the People’s Republic of China (Yang et al., 2003). Also, in Iranian women, breast cancer is considered the most common malignancies especially in younger women (Akbari et al., 2017). 

The pathologic factors of breast cancer are not well understood, though some reports referred to the genetic and environmental parameters as the causes of breast cancer (Mettlin, 1999; Sharif et al., 2016). One of the significant factors of the genetic susceptibility to cancer is the inherited differences in the potential of the xenobiotic-metabolizing enzymes. Moreover, glutathione S-transferases (GSTs) have been considered the phase II enzymes that contribute to detoxifying various toxic and potentially carcinogenic compositions (Hayes and Pulford, 1995). Researchers recognized 5 classes of GST enzymes in human (GST classes α, μ, π, σ, and θ). A separate gene or gene family encodes each of the classes. Then, the allelic variants for each gene can cause less efficient or absent enzymatic detoxification, thereby increasing susceptibility to cancer; however, we have no accurate knowledge of the biochemical procedures. One of the most common members of GSTs is the GSTP1 which has a well-known genetic variation entitled rs1695 single nucleotide polymorphism (SNP). This gene is located on chromosome 11 (11q13) and the mentioned SNP causes an amino acid substitution has been described at codon 105 (A313G→Ile105Val), resulting in the production of a functionally changed enzyme (Vogl et al., 2004; Udomsinprasert et al., 2005). Another common genetic variation for GSTP1 is the rs1138272 which results in Ala to Val substitution at codon 114 (Val114Ala). The GST class *π* gene encodes the GSTP1 enzyme is chiefly observed in the heart, spleen, and lung tissues. The GST class π enzyme is also expressed in breast cancer tissue (Kelley et al., 1994).

Most investigations found a relationship between breast cancer and *GSTP1* common polymorphisms (Liu et al., 2013; Khabaz, 2014; Jaramillo-Rangel et al., 2015; Kimi et al., 2016; Song et al., 2016). Nonetheless, these investigations did not lead to a firm conclusion. On the other hand, there are no sufficient studies investigating the association of these single nucleotide polymorphisms with breast cancer risk. Hence, this case-control study has been done to provide a relatively reliable result which is followed by a bioinformatics approach.

## Materials and Methods

*Case-control study*

*Subjects*

An attempt was made to design the project and the paper according to the rules of the STREGA (Table S1). In this case-control study, based on the number of available samples, 100 women with the mean age of 44.26±6.80 years with only sporadic breast cancer and 100 healthy women with the mean age of 45.48±6.08 years were enrolled. We chose the controls and cases from the women referring to the Pasteur pathobiology and genetics laboratory and also Rohani hospital (Babol, Iran). Diagnosing breast cancer has been proved by histological examinations for case participants. Moreover, the controls have been chosen from healthy women referring to the same hospital for routine examinations. Notably, each control participant lacked a history of oncological diseases. In addition, 3 mL of blood has been obtained from each subject. Besides, the present research has been performed according to the principles proposed in the Declaration of Helsinki.

*GSTP1 polymorphisms genotyping*

In this study, we evaluated two common SNPs in *GSTP1 *gene. According to the research design, a commercial Kit (CinnaGen, Tehran, Iran) has been used to extract genomic DNA from the blood samples. Then, the PCR strategy has been used to amplify the GSTP1 fragment containing rs1695 and rs1138272 polymorphisms. Moreover, PCR has been amplified in a final volume of 30 μl. Its mixture consisted of 1×PCR buffer, 50 ng of template DNA, 0.5 μL dNTPs mix, 2.5 μM MgCl_2_, and 2.5 U of Taq polymerase, and 0.35 μM each of forward and reverse primers (F: 5’-CTCTCATCCTTCCACGCACATCC-3’ and R: 5’-CTGCACCCTGACCCAAGAAGGG-3’ for rs1695 and F: 5’-ACAGGATTTGGTACTAGCCT-3’ and R: 5’-AGTGCCTTCACATAGTCATCCTTG-3’ for rs1138272). All PCR reagents were ordered from CinnaGen Company (Tehran, Iran). PCR procedure was done in an Eppendorf thermal cycler (Eppendorf AG, Hamburg, Germany). Notably, PCR amplification started with an early denaturation at 94°C for five minutes and then 33 cycles with 94°C (45 sec), 60°C (45 sec) and 72°C (45 sec) have proceeded. In addition, the final extension has been done at 72°C for 10 min, and thus the cycle ended to maintain at 4°C. The PCR products were treated with BsmAI restriction enzyme (Fermentas, Germany) for rs1695 and AciI (Fermentas, Germany) for rs1138272 according to manufacturer’s instruction. Finally, the PCR-RFLP products have been electrophoresed on 1.5 % agarose stained with Green Viewer™. UV transillumination has been used to visualize the amplicon bands. For rs1695 SNP, the samples with one band (363-bp) on 2.0% agarose gel had genotype AA while the samples with two bands (226- and 137-bp) on agarose gel had genotype GG. Therefore, the sample containing three mentioned fragments (363-, 226- and 137-bp) were considered as heterozygote genotype AG. Regarding rs1138272 polymorphism, the fragment with allele C was digested to two fragments (143- and 27-bp) and allele T has no restriction site for the enzyme (170-bp) that was detected on 3.0% agarose gel.

*Statistical analyses*

According to the research design, the Chi-squared test has been applied for evaluating the Hardy-Weinberg equilibrium (HWE). Moreover, a p-value less of than 0.05 has been considered as a significant deviation from the HWE. Then, in the present case-control study, we computed OR with 95% CI for all genotypes and alleles in the cases and controls. Additionally, a chi-square test has been used to evaluate the differences between cases and controls (Rafatmanesh et al., 2018; Talebi et al., 2018). Then, a two-tailed p-value<0.05 has been regarded as statistically significant. The SPSS 20 (SPSS Inc., IBM Corp Armonk, NY, USA) has been used to run the statistical analysis.

*In silico analysis*

In this study, we evaluated the molecular effects of rs1695 polymorphism on the *GSTP1* structure by the SNPeffects web server. For this purpose, at first, the entire sequence of the *GSTP1* gene was obtained from NCBI. Then the coding sequence of the gene was deduced and then was translated to amino acid sequence by ExPASy bioinformatics webserver. The location of the rs1695 variation was determined on codon 105 (Ile105Val). This sequence was introduced to SNPeffects online web server and then the influence of rs1695 SNP on aggregation propensity (TANGO), amyloid propensity (WALTZ) and chaperone binding (LIMBO) was evaluated (De Baets et al., 2011).

## Results

*Genetic association outcomes*

For, rs1695 polymorphism, our data from genotypes distribution showed no deviation from Hardy-Weinberg equilibrium in our study for both case and control groups. The distribution of alleles and genotypes of rs1695 polymorphism are summarized in Table 1. After analysis, we found that the frequency of genotypes AA, AG, and GG for controls is 50.00%, 44.00%, and 06.00%, respectively. While these ratios were calculated 37.00%, 49.00%, and 14.00%, respectively. The statistical analysis revealed that the homozygote genotype GG is associated with increased risk of breast cancer (OR= 3.1532, 95%CI= 1.1072 to 8.9798, p= 0.0315) but AG genotype was not associated with the risk of breast cancer (OR= 1.5049, 95%CI= 0.8354 to 2.7109, p= 0.1734). Although carriers of allele G had high frequency in the patient population, this difference was not statistically significant. Moreover, allele analysis revealed that the allele G is associated with the increased risk of breast cancer (OR= 1.6098, 95%CI= 1.0577 to 2.4500, p= 0.0263).

For, rs1138272 polymorphism, our data from genotypes distribution showed no deviation from Hardy-Weinberg equilibrium for both case and control groups. The distribution of alleles and genotypes of rs1138272 polymorphism are summarized in Table 2. After analysis, we found that the frequency of genotypes CC, CT, and TT for controls is 92.00%, 08.00%, and 00.00%, respectively. While these ratios for cases were calculated 88.00%, 11.00%, and 01.00%, respectively. The statistical analysis revealed that the homozygote TT (OR= 3.1356, 95%CI= 0.1260 to 78.0014, p= 0.4858) and heterozygote CT (OR= 1.4375, 95%CI= 0.5524 to 3.7411, p= 0.4571) genotype are not associated with risk of breast cancer. Also, the carriers of allele T had no high frequency in the patient population compared to controls. Moreover, allele analysis revealed that the allele T is not associated with the risk of breast cancer (OR= 1.6684, 95%CI= 0.6760 to 4.1180, p= 0.2668).

*In silico analysis*

**Figure 1:**
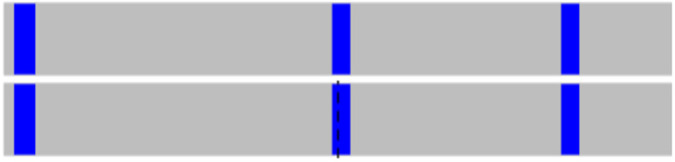
Bar Depiction of the WALTZ Windows Exist in the Normal (up) and Variant GSTP1 (down). The location of the aggregating stretches is pictured in blue color, and the رertical dotted line in the variant shows the location of the mutant residue

**Figure 2:**
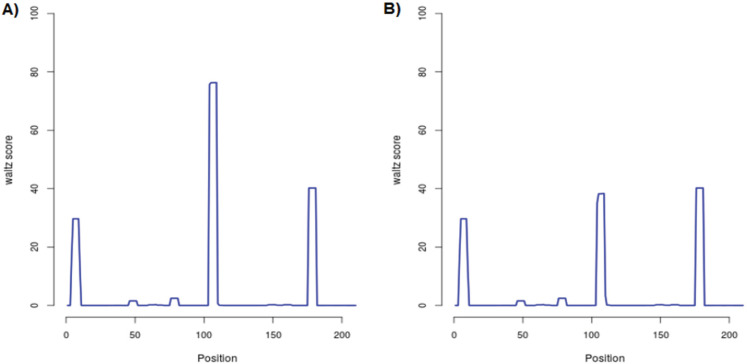
Profile Depiction of the WALTZ Stretches in the Normal Type (A) and Variant (B) Enzyme. This graph schemes the per-residue WALTZ aggregation value of the normal type and mutant enzyme. All residue values from the N-terminal to the C-terminal are planned from left to right

**Figure 3:**
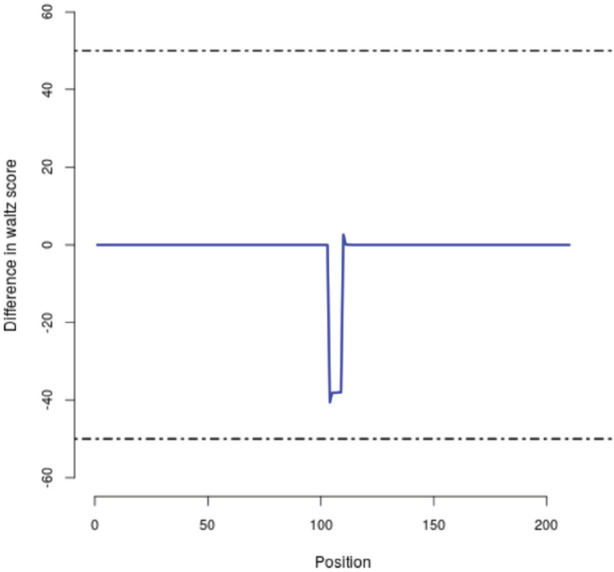
The Difference in WALTZ Amyloid Propensity between Wild Type and Mutant Protein. This diagram plans the difference of per-residue WALTZ aggregation value between normal GSTP1 and the mutant. All WALTZ value differences from the N-terminal to the C-terminal are planned from left to right. A horizontal line shows that the SNP does not change the aggregation profile of the GSTP1. Positive peaks show elevated amyloid propensity due to this SNP. Negative peaks show reduced amyloid propensity due to this SNP

We evaluated the effects of Ile105Val single nucleotide polymorphism on the *GSTP1* function by SNPeffects online software. Our data revealed that based on the TANGO model, the Ile105Val SNP does not influence the aggregation tendency of our studied protein as well as LIMBO analysis, the mentioned SNP does not affect the chaperone binding tendency of GSTP1 protein. But, based on WALTZ output, we found that the Ile105Val polymorphism decreases the amyloid propensity of the GSTP1 enzyme. WALTZ algorithm specifically and accurately predicts amyloid-forming areas in the sequences of protein. The overall WALTZ value for CSTP1 estimated 906.54 and mutations/variations could elevate (dWALTZ >50), reduce (dWALTZ <-50) or not influence amyloid propensity (dWALTZ between -50 and 50). In our analysis, dWALTZ was calculated -228.00 which means that the polymorphism reduces the amyloid propensity of the *GSTP1*. In Figures 1 and 2, the location of the WALTZ stretches in the normal type and mutant enzyme are shown, demonstrate by correspondingly a bar or profile illustration. The short stretches are recorded for both mutant and wild type (Table 3). To compare the influence of the SNP to the wild type, we also display a Difference outline (Figure 3), that plans the difference between wild and mutant protein.

**Table 1:** Association Analysis of rs1695 with Breast Cancer Risk

Genotype/Allale	No. and Percentage		OR (95% CI)	*P*-value
	Controls (n=100)	Cases (n=100)		
AA	50 (50.00%)	37 (37.00%)	-	-
AG	44 (44.00%)	49 (49.00%)	1.5049 (0.8354 to 2.7109)	0.1734
GG	6 (06.00%)	14 (14.00%)	3.1532 (1.1072 to 8.9798)	0.0315
GG+AG	50 (50.00%)	63 (63.00%)	1.7027 (0.9684 to 2.9938)	0.0645
A	144 (72.00%)	123 (61.50%)	-	-
G	56 (28.00%)	77 (38.50%)	1.6098 (1.0577 to 2.4500)	0.0263

OR, odds ratio; CI, confidence interval; Significant difference between patient and control groups are bolded

**Table 2.** Association Analysis of rs1138272 with Breast Cancer Risk

Genotype/Allale	No. and Percentage		OR (95% CI)	*P*-value
	Controls (n=100)	Cases (n=100)		
CC	92 (92.00%)	88 (88.00%)	-	-
CT	8 (08.00%)	11 (11.00%)	1.4375 (0.5524 to 3.7411)	0.4571
TT	0 (00.00%)	1 (01.00%)	3.1356 (0.1260 to 78.0014)	0.4858
CT+TT	8 (08.00%)	12 (12.00%)	1.5682 (0.6119 to 4.0191)	0.3488
C	192 (96.00%)	187 (93.50%)	-	-
T	8 (04.00%)	13 (06.50%)	1.6684 (0.6760 to 4.1180)	0.2668

OR, odds ratio; CI, confidence interval

**Table 3:** WALTZ Areas in Wild and Mutant Types. For each WALTZ area, the start, end, peptide sequence and score is detailed

Number	Start	End	Stretch	Score
Wild type				
1	3	10	YTVVYFP	25.45
2	103	109	YISLIY	76.32
3	175	181	LLSAYV	40.21
Mutant				
1	3	10	YTVVYFP	25.45
2	103	109	YVSLIY	38.28
3	175	181	LLSAYV	40.21

## Discussion

It has been found that allelic variability at a single locus could not explain the etiology of a majority of the common cancers. Besides, a main load of cancer in the general population likely is the result of the complicated interaction of several environmental and genetic parameters during the time. However, perception of the interactions of endogenous physiology, xenobiotic exposure, and genetic variability at numerous loci would provide information about the cancer etiology and detect people at the greater risks of progressing cancer. In this study, we evaluated the association of two common genetic variations (Ile105Val and Val114Ala) in the *GSTP1* with breast cancer risk. This enzyme is a member of enzymes family that has an essential role in detoxification by catalyzing the conjugation of various electrophilic and hydrophobic compounds with reduced glutathione. Our data revealed that Ile105Val variation as an exonic SNP could alter the risk of breast cancer. In detail, the genotype homozygote GG is associated with the risk of breast cancer. Also, allele analysis revealed a true association of allele G and breast cancer susceptibility. However, we did not find any significant association between rs1138272 polymorphism and breast cancer risk. Some studies were investigating the association of the *GSTP1* polymorphisms with breast cancer risk. For example, Samson et al., (2007) reported a non-significant elevation in the risk of breast cancer was observed among women who had the *GSTP1* Val/Val genotype (Samson et al., 2007). While, Ge et al., (2013) reported a positive association between *GSTP1*-Ile105Val polymorphism and breast cancer risk (Ge et al., 2013). The dissimilar results from different studies may be due to environmental, geographic, race, and other factors.

Notably, the conjugation or addition of the aliphatic-aromatic heterocyclic radicals, epoxide, or arene oxide to glutathione is catalyzed by the mentioned enzymes (Kemper et al., 2014). Moreover, the conjugation reaction at the electrophilic center of such compositions happens at the sulfur atom of the glutathione molecule (Rebbeck, 1997). Therefore, the molecules function as the glutathione peroxidase; however, they did not need a selenium co-factor for performing the conjugation reaction (Rebbeck, 1997). Also, *GSTP1* is a gene that is related to DNA repair, and keeps DNA from an impairment, controls detoxification and metabolism, so preventing tumor incidence. The *GSTP1* gene methylation often shows tumors progression, including breast cancer or unfavorable prognosis (Schnekenburger et al., 2014). Consistent with the role of methylation of GSTP1 in the tumor’s progression, a study showed significantly increased gene methylation of GSTP1 in breast cancer cells, which was positively associated with tumor size and TNM stage, and negatively associated with the expression of ER/PR (Schnekenburger et al., 2014). This evidence could elucidate the main role of disrupted GSTP1 in breast susceptibility. This could explain the role of key single nucleotide polymorphisms in the pathogenesis of GSTP1. Genetic variations based on their positions in a gene could alter the gene function (Salimi et al., 2017; Nejati et al., 2018). The SNPs on the promoter of a gene may alter the gene expression however the SNPs in the intron regions could alter the production of mature mRNA by interfering with the splicing process (Mobasseri et al., 2019; Zamani-Badi et al., 2019). But, the missense mutations could alter the structure and function of proteins (Noureddini et al., 2018; Bafrani et al., 2019) what may be true for *GSTP1*-Ile105Val genetic variation. Evaluation of the impacts of genetic variations by biological experiments is a very difficult process and evaluation of these impacts could be much easier with the in silico tools (Tameh et al., 2018; Zamani-Badi et al., 2018). In this study, also we employed the SNPeffects bioinformatics tool to evaluate the molecular effects of Ile105Val SNP on the *GSTP1* gene and we found that this polymorphism decreases the amyloid propensity of GSTP1 protein and therefore, the pathogenic effect of Ile105Val may arise from this issue.

In addition to *GSTP1*, recently, some reviews (Armstrong, 1991; Guengerich et al., 1992; Daniel, 1993) illustrated the protein structure, inducibility, enzymology, and level of expression (e.g., gender- and tissue-specific expression) of *GSTTl* and *GSTM1*. They provided a summary of *GSTTl* or *GSTM1* contribution to the metabolisms of and induction by multiple popular or uncertain carcinogenic compounds. The compounds are benzo-(a)pyrene, styrene-7,8-oxide, and trans-stilbene oxide by *GSTM1* and epoxybutanes, halomethanes, ethylene oxide, and methyl bromide by *GSTT1*. According to the above list, we do not observe a single class of chemical compositions, which has been related to the *GSTM*1 or *GSTT* induction or metabolism (Hayes and Pulford, 1995). Finally, it has been found that *GSTTl* and *GSTM1* contribute to the metabolisms of multiple xenobiotics like the chemotherapeutic factors, environmental carcinogens, and reactive oxygen samples (Tew et al., 1993). Besides, it seems that *GSTM1* distinctly involves the susceptibility to cancer including breast cancer due to its possible distinctive substrate qualities.

Our study showed that *GSTP1*-Ile105Val polymorphism could be a genetic risk factor for breast cancer. Based on this theory, the mentioned polymorphism could be considered as a biomarker for screening of susceptible women. However, there are some limitations in our study which should be mentioned. At first, we did not evaluate the influence of gene-gene and gene-environmental factors. Also, the small sample size of our study could be considered as the second limitation of our study. Therefore, evaluation of this polymorphism in larger sample size with regard to the aforementioned interactions could result in more accurate outcomes.
